# Supplementary material for: Is There Any Difference in the In Situ Immune Response in Active Localized Cutaneous Leishmaniasis That Respond Well or Poorly to Meglumine Antimoniate Treatment or Spontaneously Heal?
Source: Microorganisms. 2023 Jun 22;11(7):1631. doi: 10.3390/microorganisms11071631 (PMC10384164; doi:10.3390/microorganisms11071631)
Supplement: Supplementary file 1 [file microorganisms-11-01631-s001.zip › microorganisms-2311054-supplementary.pdf]

Comparision of PRL1 and PRL2 markers by Wilcoxon test.

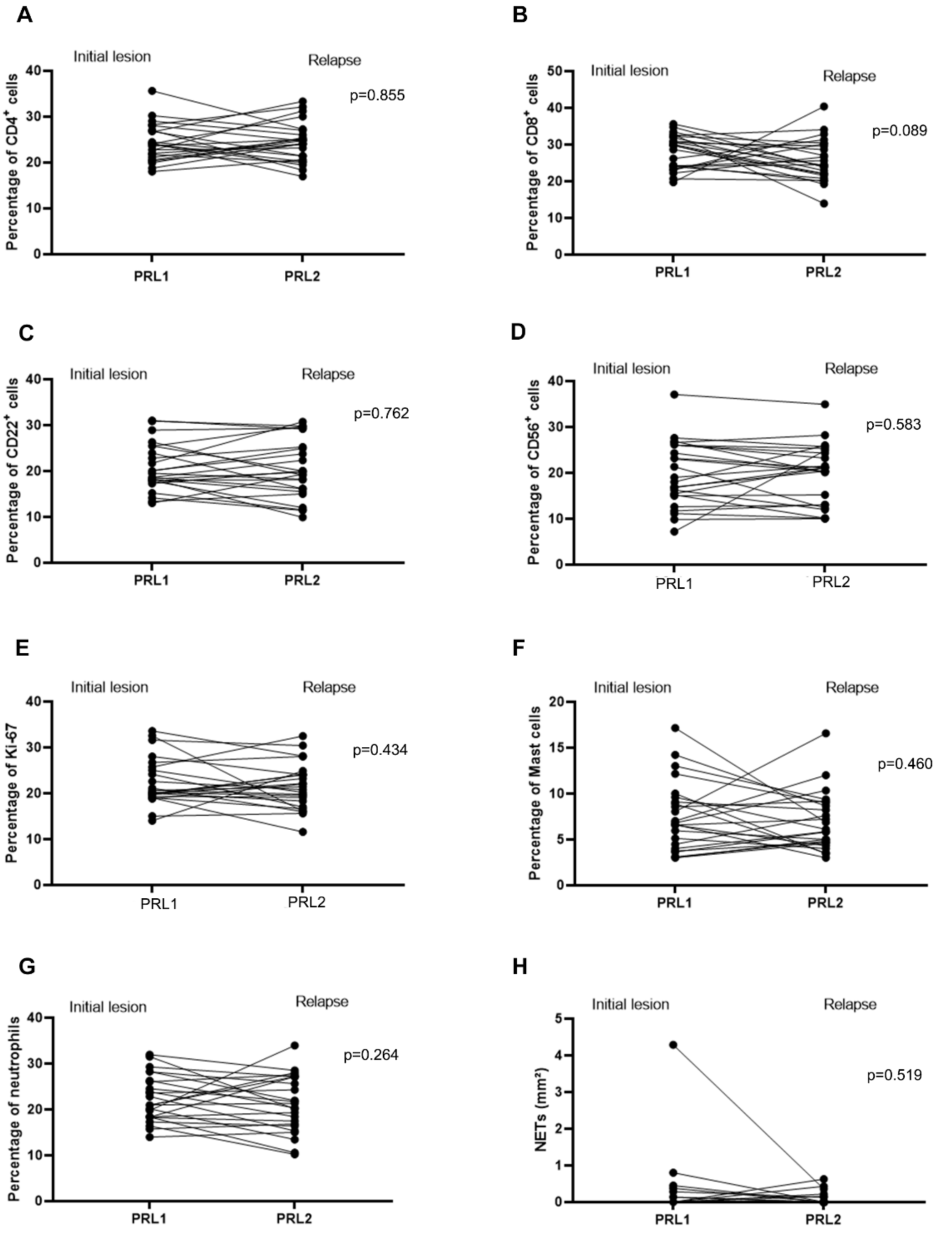

Supplementary Figure S1- Percentage of **(A)** CD4+**,** **(B)** CD8+ **(C)** CD22+, **(D)** CD56+, **(E)** Ki-67, **(F)** Mast cells, **(G)** Neutrophils and **(H)** NETs in PRL patients. Each line corresponds to one patient. Wilcoxon test. Data represented as mean and SEM. P value ≤0.05 was considered statistically significant. PRL1: non-responders to the specific treatment (initial lesion) and PRL2: non responders (relapse).

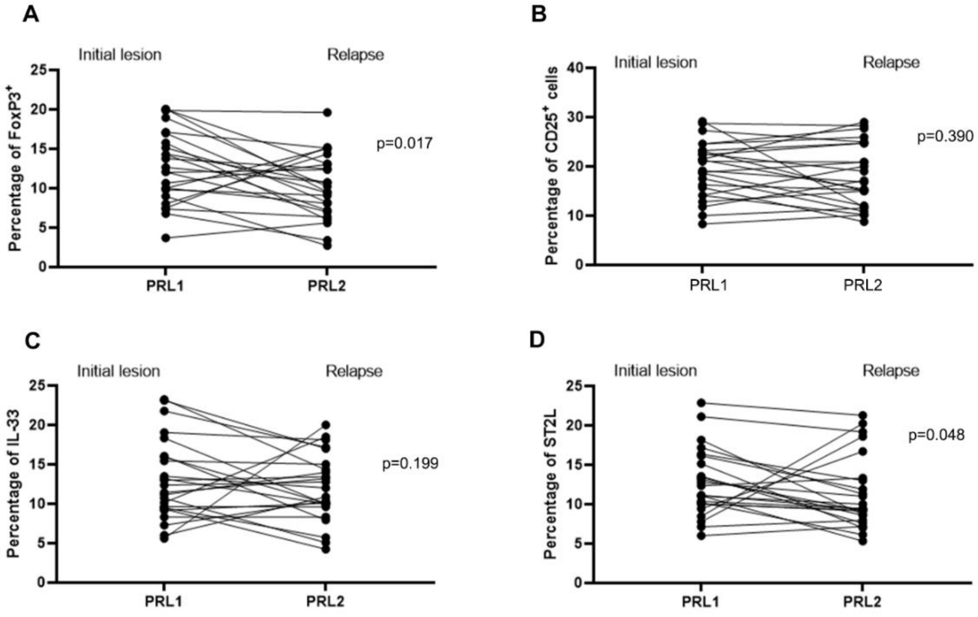

Supplementary Figure S2. Percentage of **(A)** FoxP3+**, (B)** CD25+ **(C)** IL-33 and **(D)** ST2L in PRL patients. Each line corresponds to one patient. Wilcoxon test. Data represented as mean and SEM. P value ≤0.05 was considered statistically significant. PRL1: non-responders to the specific treatment (initial lesion) and PRL2: non-responders (relapse).

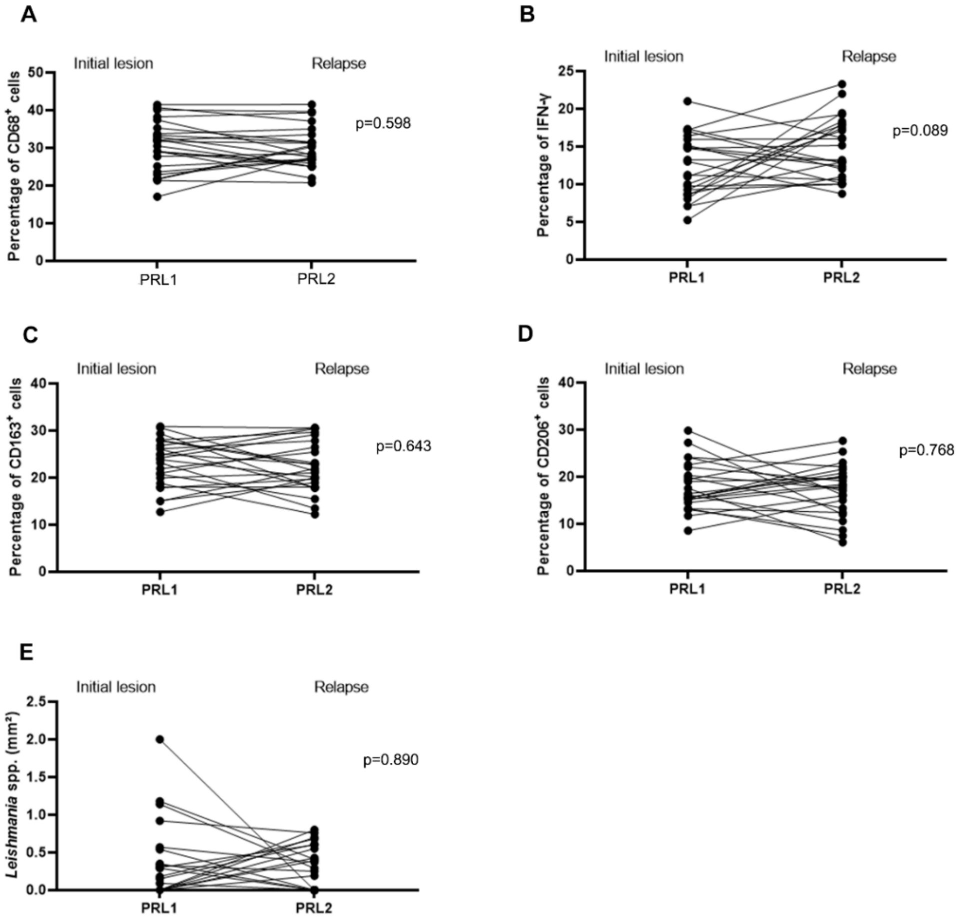

Supplementary Figure S3. Percentage of **(A)** CD68+**, (B)** IFN-y **(C)** CD163+, **(D)** CD206+ and **(E)** *Leishmania* spp in PRL patients. Each line corresponds to one patient. Wilcoxon test. Data represented as mean and SEM. P value ≤0.05 was considered estatistically significant. PRL1: non-responders to the specific treatment (initial lesion) and PRL2: non-responders (relapse).
